# Supplementary material for: Perceptions of receiving behaviour change interventions from GPs during routine consultations: A qualitative study
Source: PLoS One. 2020 May 21;15(5):e0233399. doi: 10.1371/journal.pone.0233399 (PMC7241720; doi:10.1371/journal.pone.0233399)
Supplement: S1 File — (DOCX) [file pone.0233399.s001.docx]

**Interview topic guide***

| **General perceptions of GPs providing heathy lifestyle advice to people to during routine consultations** |
| --- |
| 1. What do you think about Making Every Contact Count (MECC) in general?   *Interviewer explains briefly the principles behind MECC (healthcare professionals advised to talk to people about health behaviour change at every possible opportunity)*   - Have you heard of this before? Do you think it is done well by GPs? Why/why not? |
| 1. How easy do you think it is for GPs to engage people in conversations about health behaviour (e.g. being more physically active, cutting down your alcohol intake)? 2. What impact do you think it has on people? |
| **Role responsibility for MECC** |
| 1. What role do GPs play in talking about health behaviour? 2. Do you see this as something GPs are personally responsible for? 3. What do you think are the benefits of GP talking to people about healthy living? 4. What do you think are the harms of GPs not initiating discussions about healthy living with people? |
| **Experiences of attending GP appointments** |
| 1. Talk me through a time where you have visited a GP for a routine appointment. Was health behaviour discussed?  - If yes, how did you feel about this experience? Was it successful? - If no, why do you think it was not discussed? |
| 1. Could you tell me about when a specific health behaviour was discussed?    - *Probes for interviewer:*       - What information did they give to you, what did they ask you to do, any tips they gave you to achieve this, what advice they gave you? |
| 1. How **appropriate** do you think it is for GPs to be discussing health behaviour with you? 2. How **helpful** do you think it is for GPs to be discussing health behaviour with you? 3. To what extent do you **expect** GPs to discuss health behaviour with you? |
| Is there anything that we haven’t covered that you feel is important or relevant? |

*Topic guide will be developed/modified iteratively depending on participants’ responses to the questionnaire
