# Supplementary material for: Perceptions of receiving behaviour change interventions from GPs during routine consultations: A qualitative study
Source: PLoS One. 2020 May 21;15(5):e0233399. doi: 10.1371/journal.pone.0233399 (PMC7241720; doi:10.1371/journal.pone.0233399)
Supplement: Attachment — Submitted filename: Response to reviewer comments_April 2020_Final.docx [file pone.0233399.s004.docx]

Response to reviewer comments

| Comment | Response | Specific changes |
| --- | --- | --- |
| Abstract cites 4 major themes, but only 3 are outlined | Thank you for highlighting this error; this has been amended in the revision. | Changes made. |
| I think they should revise the claim that this is the "first" investigation of its type in the field of behaviour change research. Although it is an interesting study and of interest, it adds to an existing body of work. For example (based on a quick Google search)  <https://bmcobes.biomedcentral.com/track/pdf/10.1186/s40608-018-0184-6?site=bmcobes.biomedcentral.com>  <https://trialsjournal.biomedcentral.com/articles/10.1186/1745-6215-14-393>  <https://bjgp.org/content/68/674/e646.short>  <https://journals.plos.org/plosone/article?id=10.1371/journal.pone.0212169> | Thank you for making this suggestion, and for providing some relevant citations. We agree that the relevance of this work would be better framed as adding to an existing body of work. However, it is worth noting that most of the current evidence base has focused on behaviour change in the context of an existing health condition, and not about interventions delivered during a routine GP consultation. We have cited the most relevant studies. We have also made a slight amendment to the title to make it more specific.  <https://bmcobes.biomedcentral.com/track/pdf/10.1186/s40608-018-0184-6?site=bmcobes.biomedcentral.com>  This study highlights the important role that a GP can have in facilitating the delivery of an online intervention. Whilst the study does not focus on interventions delivered within routine consultations, we agree this is an important addition to the background literature.  <https://trialsjournal.biomedcentral.com/articles/10.1186/1745-6215-14-393>  This is a protocol paper and we refer already to the main paper (Aveyard et al., 2016). We have provided additional detail for clarification.  <https://bjgp.org/content/68/674/e646.short>  This paper shows the importance of GP-patient interactions as a platform for delivering interventions. We thank the reviewer for highlighting this useful paper, and have added it to the background.  <https://journals.plos.org/plosone/article?id=10.1371/journal.pone.0212169>  In the context of CVD prevention, this paper illustrates the willingness of patients to receive an intervention in the primary care context and we have added it to the background.  We have also made some slight amendments including adding further relevant citations (highlighted in the reference list). | Page 3, lines 61-62  Research has shown that interventions facilitated by a GP in a primary care setting are effective, and are welcomed by patients [4].  Page 3, lines 64-65  Research suggests that the GP-patient interaction provides an important foundation for delivering behaviour change interventions [6-9].  Page 4, lines 81-84  Research has examined patients’ willingness to receive behaviour change interventions within specific health contexts, such as during cancer screening appointments [19] or in the context of managing or preventing long-term health conditions including psoriasis [20] and cardiovascular disease [21],  Page 4, lines 86*87  Consequently, more research is needed on the patient perspective of receiving opportunistic behaviour change interventions from GPs, during routine primary care consultations.  Page 15, lines 362-363  This study examined the perceptions of patients receiving health behaviour change interventions from a GP during routine medical consultations.  Page 17, lines 420-422  This study examined people’s perceptions of receiving behaviour change interventions during routine GP consultations. Findings strengthen the growing evidence base highlighting important opportunities for GPs  Please also see suggested change in title:  Perceptions of receiving behaviour change interventions from GPs *during routine consultations*: A qualitative study |
