# Supplementary material for: Perceptions of receiving behaviour change interventions from GPs during routine consultations: A qualitative study
Source: PLoS One. 2020 May 21;15(5):e0233399. doi: 10.1371/journal.pone.0233399 (PMC7241720; doi:10.1371/journal.pone.0233399)
Supplement: Attachment — Submitted filename: Response to reviewer comments.docx [file pone.0233399.s003.docx]

Response to reviewer comments

| Comment | Response | Specific changes |
| --- | --- | --- |
| The methods needs a framework to provide details within sections. Please selected the COREQ (Tong et al) or SRSQ (O'Brien) or another suitable one and make sure the methods addresses the points most important to this type of work. | We have included the COREQ framework as an additional file, and ensured that we have included the relevant points in the methods section. | Please see the following amendments:  We selected a purposive sample with maximum variation to obtain the widest possible views from participants in terms of age, gender and social grade (pages 4-5)  Interviews were conducted by one of the study authors (either CK or JG; both trained researchers in conducting qualitative interviews) (page 5)  The final sample (*n*=24), recruited from a total of 493 people who were happy to contacted to take part in the semi-structured interview, (page 6)  Length of interviews ranged from 15 to 45 min (mean length 25 min). (page 6) |
| Please make sure you give a methodology and a paradigmatic stance and consider the implications for this on how you have written your results and how you view quality. | We have amended the methods section for clarity and to provide extra detail on the pragmatic stance. | Please see the following amendments:  We took a critical realist stance to analyzing the data. This approach assumes that we can capture a reality that exists for the participants in specific contexts, including causal relationships, so long as a reflexive approach is maintained. Due to a lack of existing research, we used inductive thematic analysis to analyse the data. This ensured the findings were data-driven, and we were able to identify and establish patterns across the data^17^. (page 5)  One of the study authors carried out the analysis (XX) using NVivo version 12, which was used to organise and manage the data. An appropriate audit trail of the analysis was ensured using field notes^18^ after each interview to ensure that interpretations were based on the data collected. We used established quality checking procedures, including critical scrutiny and constant comparison. The lead author shared the coding framework and key illustrative quotes with the other authors as the analysis progressed. Authors discussed any areas of contention and codes and themes were refined accordingly in order to ensure trustworthiness of the data.  (pages 5-6) |
| Please consider aspects such as sampling and sample size, an audit trail within a supplementary file, trustworthiness etc and then resubmit the paper. | Our participants previously took part in a large national survey, and many of the participants agreed to be contacted for further information about the interview study. Our sampling frame enabled us to capture the widest possible variation in views, whilst ensuring we could answer the research questions. We have added extra detail outlining this process, as well as document the analysis procedure. | In addition to the changes made above, please also see the following amendments:  We selected a purposive sample with maximum variation to obtain the widest possible views from participants in terms of age, gender and social grade. (pages 4-5)  Whilst we aimed to capture the widest possible variation of views and opinions, and our sample size was deemed to be sufficient to answer our research questions, there may be additional views that were not captured in the present sample. (page 17) |
| Please ensure that your manuscript meets PLOS ONE's style requirements, including those for file naming. The PLOS ONE style templates can be found at <http://www.plosone.org/attachments/PLOSOne_formatting_sample_main_body.pdf> and <http://www.plosone.org/attachments/PLOSOne_formatting_sample_title_authors_affiliations.pdf> | We have ensured the manuscript meets PLOS ONE’s style requirements. | We have ensured the manuscript meets PLOS ONE’s style requirements. |
| Please include additional information regarding the interview guide used in the study and ensure that you have provided sufficient details that others could replicate the analyses. For instance, if you developed a guide as part of this study and it is not under a copyright more restrictive than CC-BY, please include a copy, in both the original language and English, as Supporting Information. | We have included the interview guide as a supplementary file. | We have included the interview guide as a supplementary file. |
| Please provide additional details regarding participant consent. In the ethics statement in the Methods and online submission information, please ensure that you have specified (1) whether consent was informed and (2) what type you obtained (for instance, written or verbal, and if verbal, how it was documented and witnessed). If your study included minors, state whether you obtained consent from parents or guardians. If the need for consent was waived by the ethics committee, please include this information. | We have provided extra detail regarding participant consent. | Please see the following amendment:  Participants provided written informed consent prior to the interviews, which were audio-recorded and transcribed verbatim. (page 5) |
| Thank you for including your ethics statement:  "Ethical approval for the study was obtained from a university ethics committee (ref:2018-3662-5925).".  Please amend your current ethics statement to include the full name of the ethics committee/institutional review board(s) that approved your specific study.  Once you have amended this/these statement(s) in the Methods section of the manuscript, please add the same text to the “Ethics Statement” field of the submission form (via “Edit Submission”). | We have amended our ethics statement. | Please see the following amendment:  Ethical approval for the study was obtained from The University of Manchester Research Ethics Committee (ref: 2018-3662-5925). (page 4) |
| We note that you have indicated that data from this study are available upon request. PLOS only allows data to be available upon request if there are legal or ethical restrictions on sharing data publicly. For information on unacceptable data access restrictions, please see <http://journals.plos.org/plosone/s/data-availability#loc-unacceptable-data-access-restrictions>.  If there are ethical or legal restrictions on sharing a de-identified data set, please explain them in detail (e.g., data contain potentially identifying or sensitive patient information) and who has imposed them (e.g., an ethics committee). Please also provide contact information for a data access committee, ethics committee, or other institutional body to which data requests may be sent.  If there are no restrictions, please upload the minimal anonymized data set necessary to replicate your study findings as either Supporting Information files or to a stable, public repository and provide us with the relevant URLs, DOIs, or accession numbers. Please see <http://www.bmj.com/content/340/bmj.c181.long> for guidelines on how to de-identify and prepare clinical data for publication. For a list of acceptable repositories, please see <http://journals.plos.org/plosone/s/data-availability#loc-recommended-repositories>. | Please find attached our data availability statement. | Suggested data availability statement:  Illustrative quotations have been provided with the manuscript. Our ethical approval was based upon statements in the participant information sheet and accompanying consent forms that referred only to the use of anonymised quotations. Therefore publication of the full data set is not consistent with our consent statements. These restrictions are in place according to The University of Manchester Research Ethics Committee (ref: 2018-3662-5925). Requests for additional data, that meet the requirements for confidential data access, can be obtained from the Information Governance team at the University of Manchester via [information.governance@manchester.ac.uk](mailto:information.governance@manchester.ac.uk). |
| While revising your submission, please upload your figure files to the Preflight Analysis and Conversion Engine (PACE) digital diagnostic tool, <https://pacev2.apexcovantage.com/>. PACE helps ensure that figures meet PLOS requirements. To use PACE, you must first register as a user. Registration is free. Then, login and navigate to the UPLOAD tab, where you will find detailed instructions on how to use the tool. If you encounter any issues or have any questions when using PACE, please email us at [figures@plos.org](mailto:figures@plos.org). Please note that Supporting Information files do not need this step. | Not applicable as we do not have any figures in the manuscript. |  |
